# Gaussian Basis Sets for Crystalline Solids: All-Purpose Basis Set Libraries vs System-Specific Optimizations

**DOI:** 10.1021/acs.jctc.9b01004

**Published:** 2020-03-26

**Authors:** Loredana Edith Daga, Bartolomeo Civalleri, Lorenzo Maschio

**Affiliations:** Dipartimento di Chimica, Università di Torino and NIS (Nanostructured Interfaces and Surfaces) Centre, Via P. Giuria 5, 10125 Torino, Italy

## Abstract

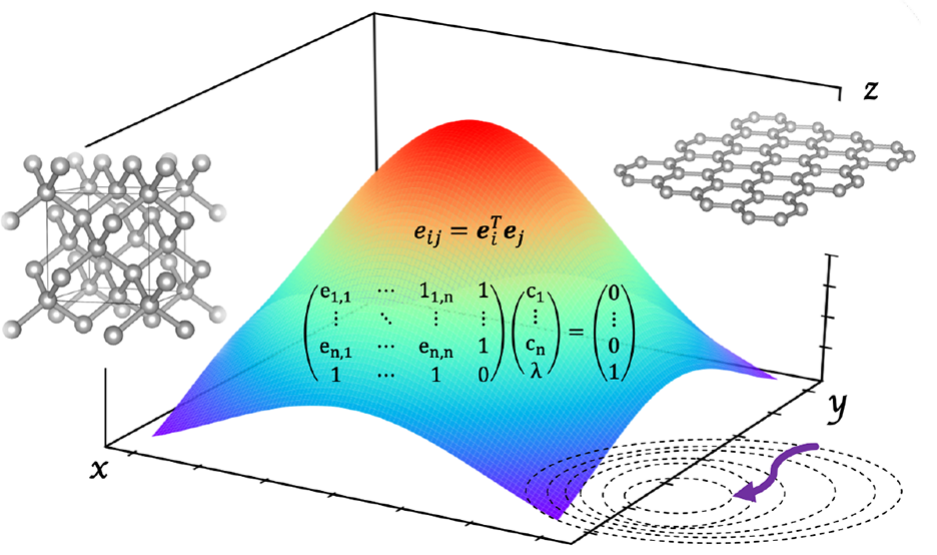

It is customary in molecular quantum chemistry to adopt basis set libraries in which the basis sets are classified according to either their size (triple-ζ, quadruple-ζ, ...) and the method/property they are optimal for (correlation-consistent, linear-response, ...) but not according to the chemistry of the system to be studied. In fact the vast majority of molecules is quite homogeneous in terms of density (i.e., atomic distances) and types of bond involved (covalent or dispersive). The situation is not the same for solids, in which the same chemical element can be found having metallic, ionic, covalent, or dispersively bound character in different crystalline forms or compounds, with different packings. This situation calls for a different approach to the choice of basis sets, namely a system-specific optimization of the basis set that requires a practical algorithm that could be used on a routine basis. In this work we develop a basis set optimization method based on an algorithm–similar to the direct inversion in the iterative subspace–that we name BDIIS. The total energy of the system is minimized together with the condition number of the overlap matrix as proposed by VandeVondele et al. [VandeVondele et al. J. Chem. Phys.2007, 227, 114105]. The details of the method are here presented, and its performance in optimizing valence orbitals is shown. As demonstrative systems we consider simple prototypical solids such as diamond, graphene sodium chloride, and LiH, and we show how basis set optimizations have certain advantages also toward the use of large (quadruple-ζ) basis sets in solids, both at the DFT and Hartree–Fock level.

## 1 Introduction

**Figure 1:**
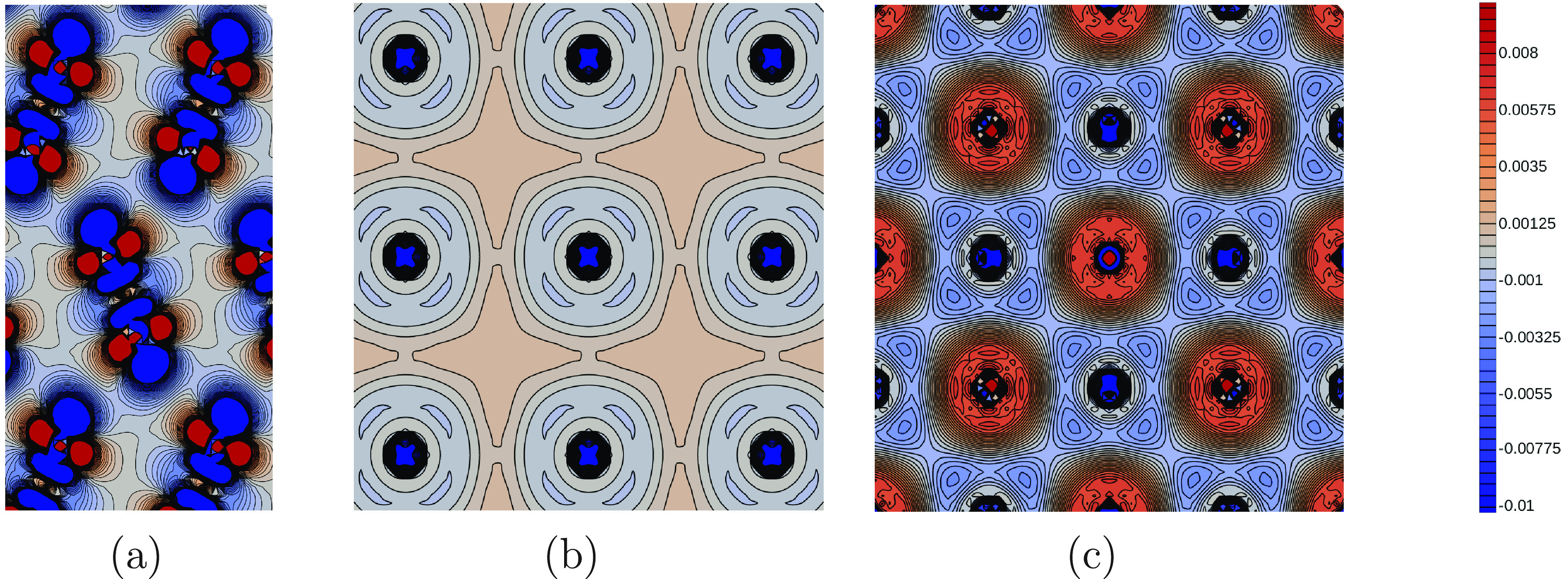
Electron difference density maps (with respect to atomic densities) of molecular solid Cl_2_ (panel a), metallic sodium (panel b), and rocksalt ionic NaCl (panel c).^37^

When dealing with the quantum chemical modeling of crystalline solids, the existence of various types of chemical bonding is clearly evident. For instance, the polymorphism of carbon in the graphite (or graphene) and diamond allotropes is just one of many examples, in which the profoundly different chemical behavior is manifested by the same chemical element in different crystal packings. Another exemplary case is that of rocksalt NaCl: sodium is by nature metallic as a bulk material, and chlorine is commonly found in the form of a molecular crystal Cl_2_. NaCl is a prototypical ionic salt. The chemical differences in those materials can be made evident by looking at their electron density (see Figure 1): the electrons involved in the metallic bond in Na are quite spread out over the whole space, while in Cl_2_ the density is somewhat more localized on molecules, with empty space between them. Conversely, the wave function in an ionic system like NaCl is strongly confined in a vicinity of the ions and features nodes in the planes in between neighboring atoms. NaCl is also considerably more densely packed.

This variety of chemical bondings in the solid state then reflects the choice of the type and quality of the basis set adopted in the mathematical form of the wave function when solving the Schrödinger equation within periodic boundary conditions (i.e., Bloch functions).^1−3^ The situation in the field of molecular modeling is somewhat simpler as isolated molecules or molecular aggregates have nearly comparable atomic densities, and there are commonly no analogue extended systems featuring metallic, ionic, or covalent bonds. Therefore, in molecular calculations, atom-centered basis sets as Gaussian-type orbitals^4^ are almost universally adopted,^5^ although other basis sets can be and are eventually used.

On the other hand, for solid-state calculations,^2^ plane waves,^6−8^ atom-centered Gaussians^9^ (or their combinations^10^), and numerical basis sets^11,12^ are all popular choices. The plane wave basis, that is naturally suited for nonlocal wave functions such as in the uniform electron gas or in a metal, has the undeniable advantage of a one-knob tuning of accuracy and cost through the kinetic energy cutoff parameter. However, the correct description of local orbitals, core states, or the void can result in a rather high computational cost. Similarly, the inclusion of exact HF exchange in hybrid HF/DFT calculations leads to a steep increase in computational time. Gaussian-type basis sets are less commonly adopted for the quantum chemical treatment of solids, with respect to plane waves. Gaussian functions have the great advantage of allowing to transfer to the solid state a large part of the technology and knowledge that is the legacy of several decades of advances in molecular quantum chemistry and to retain the chemical intuition when looking at the electronic charge distribution of the investigated system. The price to pay is the mandatory definition of a basis set for each atomic species, that is ultimately left in the hands of the end user.

Nowadays, standardized basis set libraries are not commonly available for solids as they are for molecules,^13,14^ despite recent attempts in that direction being carried out by Bredow and co-workers.^15−17^ The reasons are not only to be ascribed to a lesser effort in a systematic construction of all-purpose basis sets but also more specifically to the wide difference in chemical bonding as outlined above. First attempts to understand the role of basis functions in solids were done by Hess and co-workers,^18^ but also more recently Jensen^19^ compared atomic, molecular, and solid-state basis sets for carbon and silicon to highlight the differences originating from the different chemical environments.

Another aspect related to the adoption of Gaussian-type functions is the basis set incompleteness due to the use of a finite number of basis functions. Basis set incompleteness is an issue in all types of calculations, but most of all in calculations that employ atom-centered basis sets–Gaussians, Slater functions, or numerical orbitals. This is because the atomic basis sets can never be made complete enough in polyatomic systems, as the basis becomes overcomplete–necessitating the removal of variational degrees of freedom–before becoming complete. In molecules it is rather common to adopt a sequence of basis sets of increasing size (e.g., cc-pVXZ (X = D,T,Q,···)^20^ and pc-X (*X* = 1,2,3, ...)^21^), but this is not yet routinely applicable for solids. Therefore, reaching the basis set limit is not trivial–even for such simple systems as lithium hydride^22−26^–and is not just a matter of computational efforts: as basis sets grow larger, exponents tends to become more diffuse, linear dependency problems arise, and the convergence of infinite Coulomb and exchange series is jeopardized.

The problem of linear dependencies with an extended basis set is a matter of active research not only for solids but also for average-sized molecules.^27^ While the important role of diffuse functions in solids has been recently highlighted by Kadek et al.,^28^ too diffuse functions are often not needed for ground state calculations because of the packing of the atoms in the unit cell. Such very diffuse functions can also be added a posteriori through dual basis set techniques.^29^ Seen from another viewpoint, the main conceptual difference in basis sets meant for the solid state as opposite to molecular electronic structure calculations is that the latter have to describe the asymptotic exponential decay of the electron density in a finite system, requiring somewhat diffuse functions, whereas diffuse basis functions are generally thought not to be necessary in solid-state calculations because the density is much more uniform throughout the cell. In this work our aim is to (i) show to what extent the basis sets are different in different chemical environments, by optimizing bases of the def2-TZVP quality^30−32^ and (ii) attempt to use suitably optimized quadruple-ζ basis sets, also from the def2- family, to verify whether they can be adopted for solids without significant pruning, and outline possible strategies for reaching such goal.

To this purpose we present a technique for the optimization of basis set exponents and contraction coefficients, that is based on the Direct Inversion in the Iterative Subspace (DIIS) technique^33−35^ and actually quite similar to its geometry optimization variant, GDIIS.^36^ The algorithm is implemented in the Crystal code.^9^ We show how such optimization allows the retaining of the full number of Gaussians letting the algorithm decide about the diffuseness of the exponents.

## 2 Theoretical Framework

### 2.1 Background

In the linear combinations of atomic orbitals (LCAO) framework, the crystalline orbitals ψ are treated as linear combinations of Bloch functions (BF) ϕ that are, in turn, defined in terms of local atom-centered functions (AO) φ
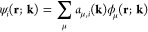
1
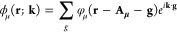
2in which **g** is a direct space lattice vector, **k** is the lattice vector defining a point in the reciprocal lattice, **A** are the coordinates of the atom in the reference cell on which the AO φ is centered, and *a* are the variational coefficients. The sum over μ is limited to the number of basis functions in the unit cell. The sum over **g** is, in principle, extended to all the (infinite) lattice vectors of the direct lattice; therefore, suitable screening techniques have to be adopted.^1,38,39^

As usual, the AOs can be written as a contraction of a number of primitive Gaussian-Type Functions (GTF) *G* centered on the same atom
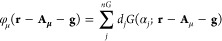
3in which *d*_*j*_ are the contraction coefficients, and α_*j*_ are the exponents of the radial component of the function. The number, type, and contraction scheme of the Gaussian basis set define its quality. Gaussian functions are defined as
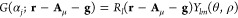
4where *R*_*l*_(**r**) = *Ne*^–α·**r**^2^^ is the radial part–*N* being a normalization constant–and *Y*_*lm*_(θ, ρ) is a spherical harmonic.

### 2.2 The BDIIS Method

Our goal is to devise a suitable algorithm for a system-specific optimization of the exponents α_*j*_ and contraction coefficients *d*_*j*_ as in eq 3. Taking inspiration from the well-known Direct Inversion of Iterative Subspace (DIIS) algorithm of Pulay,^33,34^ we describe in the following our Basis-set DIIS (BDIIS) method.

The idea is that of an iterative procedure in which, at each step *n*, exponents and contraction coefficients are obtained as a linear combination of the trial vectors obtained in previous iterations
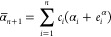
5
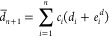
6

In the above, *e*_*i*_^α^ and *e*_*i*_^*d*^ are, respectively, the changes in exponents and contraction coefficients, as predicted by a simple Newton–Raphson step. In fact the gradients *e*_*i*_ are defined by
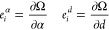
7where Ω is a suitable functional to be minimized. Here we decide to minimize the system’s total energy to which we add a penalty function including the Overlap matrix condition number, following the proposal of VandeVondele and Hutter:^40^
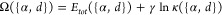
8

The value γ = 0.001 was adopted as suggested in ref (40). In eq 8, κ({α, *d*}) is the condition number, i.e., the ratio between the largest and the smallest eigenvalue of the overlap matrix at the center of the Brillouin zone (Γ-point). The purpose of such penalty function is to prevent the onset of harmful linear dependence. Linear dependence issues can give rise to numerical instabilities and, as a consequence of that, the appearance of unphysical states. Such unphysical states generally lead to a catastrophic behavior of the total energy that can drop to a value that is orders of magnitude larger, in absolute value, than the proper one.

Although the first of derivatives in (7) could be in principle computed analytically,^41,42^ in the present work we evaluate both *e*_*i*_^α^ and *e*_*i*_^*d*^ by means of numerical derivatives (*vide infra*). The length of the estimated Newton steps represented by the **e**^α^ and **e**^*d*^ can assume the meaning of an estimated distance from the minimum of Ω and thus be utilized as a measure of the “error” at step *n*.

The DIIS error matrix, that has the size of the iterative space considered, is built from the scalar products
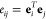
9

By imposing the constraint , we can obtain the linear combination coefficients of the BDIIS method to be used in (5) and (6) by solving the linear equation system
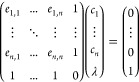
10where λ is a Lagrange multiplier. Such an approach is, in fact, similar to geometry-optimization DIIS (GDIIS^36^) adopting an identity Hessian.

### 2.3 Details of the Implementation

The BDIIS procedure outline above has been implemented in a development version of the Crystal17 code.^9^ As already mentioned, in this work we compute the derivatives in (7) by means of a two-sided numerical derivative. Which means, for exponents α
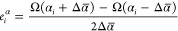
11and similarly for coefficients *d*.

The displacement is 1% of the exponent value (Δα̅ = 0.01·α), while for coefficients the step is set to 0.1%, weighted by the relative exponents (Δ*d̅* = 0.001·*d*/α).

We have also tried to compute a diagonal Hessian using the three points α_*i*_ + Δα̅, α_*i*_ and α_*i*_ – Δα̅, so to improve the step (error) as defined in eqs 5 and 6 at the same computational cost. However, such a diagonal Hessian seemed not to improve on the quality of the step, and the overall convergence pattern turned out to be similar or slower in all cases we tested. We surmise that the cause can reside in the insufficient accuracy of a three-point numerical estimate of the second derivative.

Once a suitable step Δα̅_*n*_ = α̅_*n*_ – α_*n*–1_ is obtained from eq 5, a line search is performed for tuning the optimal parameter *f*_*l*_
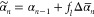
12by sampling *f*_*l*_ from 0.1 to 1 in a suitable discrete point grid. The point with the minimum value of Ω is then retained.

The convergence of the iterative optimization procedure is verified by checking the absolute value of the largest component of both the gradients and the penalty function. The iterative space used in the BDIIS procedure is set at most to the 14 previous cycles, and the BDIIS step is active since the second basis set optimization step. The optimization is complete when the absolute value of the difference in the penalty function is less than 1.0 · 10^–5^ au and the absolute value of the largest component of gradient converges to 3.0 · 10^–4^.

## 3 Results

In this section we first briefly describe the performance of the BDIIS method in minimizing the Ω energy functional as defined in eq 8. Then we focus on the effect of system-specific basis set optimizations, by showing the differences between optimized exponents of a typical triple-ζ basis in simple systems containing the same atoms but in a different chemical bonding situation. Finally, we analyze how extended basis sets, such as molecular quadruple-ζ quality, can be optimized for dense solids without significant pruning. In the Supporting Information the reader can find full Crystal17(9) inputs for all the calculations presented in the following, including the explicit definition of the atomic basis sets.

All of the optimizations in this work have been carried out starting from molecular def2-TZVP or def2-QZVP basis sets.^30−32^ Although the implemented algorithm is general, as described in the previous section, in the following we will focus on the optimization of valence and polarization functions only–the ones relevantly changing in a different chemical environment. Since they are usually uncontracted Gaussian functions, the optimization has been performed solely for the exponents. As a general strategy, we took as a starting point the molecular basis sets, upscaled the exponents of all outermost functions so to avoid small values (<0.1) but without pruning the basis set, and finally optimized the corresponding values by minimizing the function Ω.

For all the calculations the convergence for the self-consistent field (SCF) algorithm is achieved when the energy difference is 1.0 · 10^–8^ au (1.0 · 10^–10^ au for LiH) using a Monkhorst–Pack (MP) shrinking factor of 8 (64 for graphene). For triple-ζ basis sets the truncation criteria of Coulomb and Exchange infinite sums are [8,8,8,12,24] for diamond, [8,8,8,15,30] for LiH, and [8,8,8,8,16] for the other systems. Convergence in the case of quadruple-ζ basis sets requires tighter thresholds, up to [10,10,10,35,175] in the case of diamond (Hartree–Fock). In the SI we report all Crystal17 inputs that can be used to reproduce our results.

In many cases, we adopted pure GGA functionals such as PBE^43^ and PBEsol^44^ in order to have a faster time to solution. In other cases we used PBE0^45^ or Hartree–Fock. More generally, we do not regard our basis set optimizations to be much dependent on the chosen method,^46^ since we do not deal with the reoptimization of the core. As the focus of our work is on accuracy and numerical stability, we will not present timings.

### 3.1 Performance of the BDIIS Method

**Figure 2:**
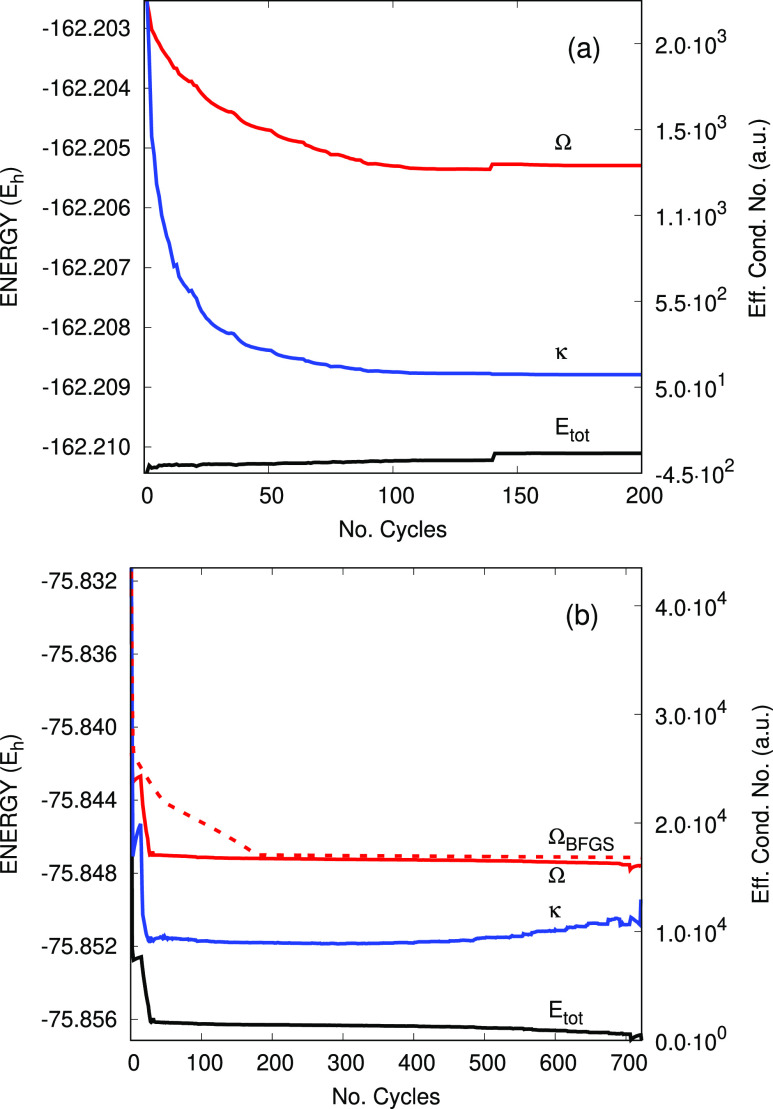
Minimization of the Ω functional of eq 8 as a function of the BDIIS algorithm iteration for two of the systems studied in this work: panel (a) sodium (PBE0), panel (b) graphene (PBEsol). The two components of the functional, E_*tot*_ and κ, are also reported individually. The dashed line in the bottom panel reports the behavior of the BFGS algorithm.

In Figure 2 we report the progress of the Ω functional minimization–cf. eq 8–along with the BDIIS iterations, in two exemplary yet challenging cases for Gaussian-type basis sets: graphene and bulk metallic sodium. In graphene, the basis set optimizer, run with the PBEsol functional, leads to a stable result after a few iterations, which represents a significant energy gain with respect to the starting point and remains stable for long. If the optimization is allowed to continue for hundreds of cycles, a rise in the penalty function γ ln κ({α,*d*}) is observed, which evidently prevents the gaussians to become too diffuse. A corresponding decrease of the electronic energy is observed. We remark that such changes are however minimal with respect to the effect of the first iterations, and the optimization is essentially converged after 50 cycles to all practical purposes. In the same figure we have also reported the curve obtained using the Broyden–Fletcher–Goldfarb–Shanno (BFGS) method. It is seen that such a method reaches the same value of the Ω functional, more slowly but also more stably. We will discuss the differences in the solution in the following.

The case of bulk metallic sodium (Figure 2(a)) is different: the electronic energy varies little (and even increases slightly with respect to the starting point); but the penalty function is much more relevant than in other cases, and about 100 iterations are required to reach a plateau. Notably, in this case the basis set optimization was carried out with a hybrid HF/DFT functional (i.e., PBE0). This level of theory is usually expected to be problematic for metallic systems, but the BDIIS algorithm runs smoothly to convergence.

### 3.2 Role of the Chemical Environment

We compare here two sets of systems, composed by the same elements: first crystalline diamond, graphene, and carbyne chain and then NaCl with bulk Na and Cl solids. We compare our system-dependent optimized basis sets with the pob-TZVP^15,17^ ones. These were also derived from def2-TZVP but differently from ours: (i) the valence exponents were optimized for each system in a comprehensive set of solids with different chemical environments, (ii) for multiple optimization of the same atomic species an averaged value of the exponent was considered, and (iii) most notably, many of the outermost functions were removed, thus reducing the consistent quality of the basis.

We will refer to the basis sets optimized in this work as “dcm-TZVP”. Since different basis sets of the same nominal quality are obtained through optimization on different systems, we will adopt the more detailed notation dcm[···]-TZVP, specifying in square brackets the system used for the optimization (e.g., dcm[NaCl]-TZVP).

#### 3.2.1 Diamond, Graphene, and Carbyne Chain

Diamond and graphene are two allotropes of carbon. Both are covalently bound systems but differ by hybridization (*sp*^3^ and *sp*^2^), as well as crystalline (3D Vs 2D) and electronic (insulator and conductor) structures. Carbyne is a model system with 1D periodicity (polymer), two atoms in the unit cell and alternating bond length.

**Figure 3:**
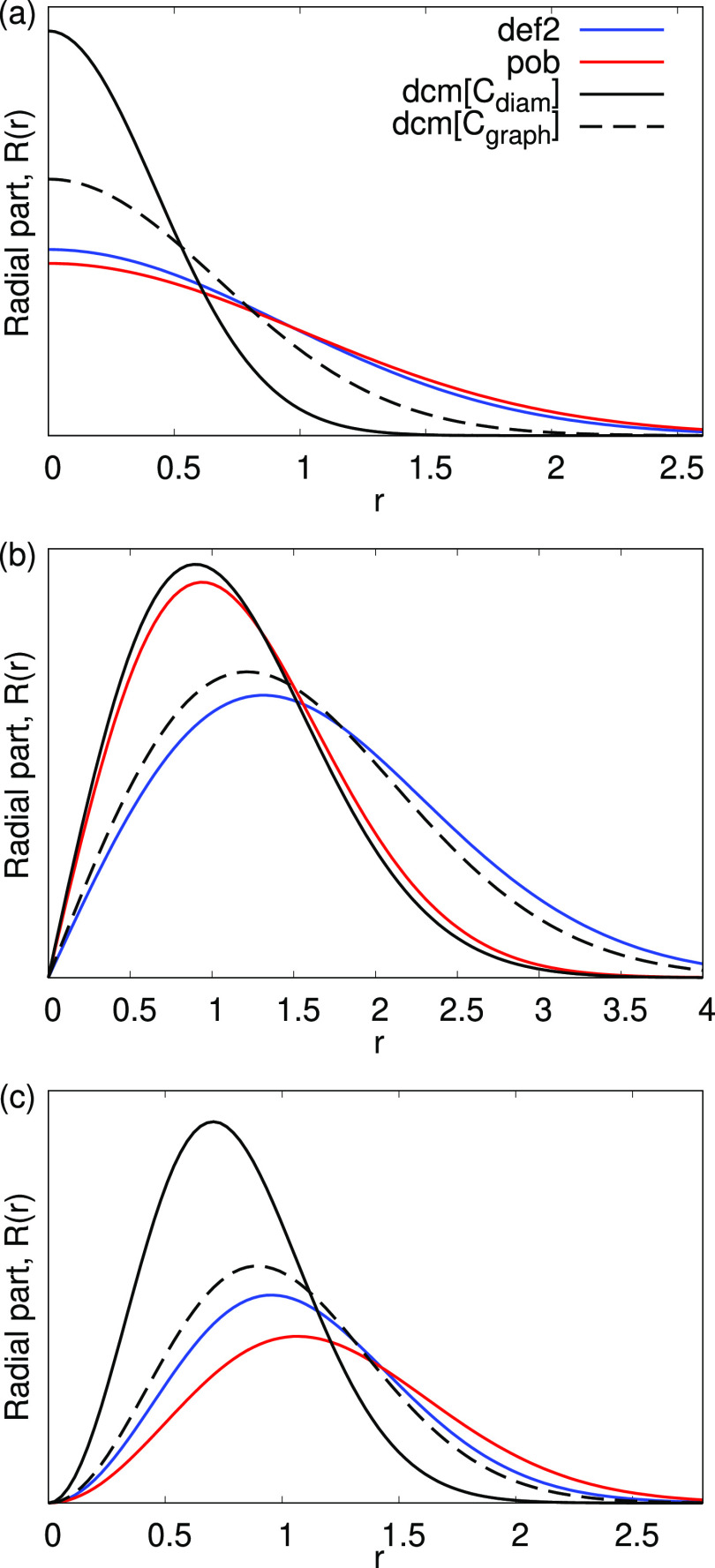
Radial part of some Gaussian functions of carbon from def2-TZVP (def2), pob-TZVP (pob), and two different dcm-TZVP (dcm[*C*_*diam*_] for diamond, dcm[*C*_*graph*_] for graphene) basis sets. Exponents of *s*-, *p*-, and *d*-type functions are reported in panels (a), (b), and (c), respectively.

In Table 1 we compare the exponents of the original def2-TZVP, the original pob-TZVP, the recently revised pob-rev2 basis set, and our dcm-TZVP basis specifically optimized for diamond, graphene, and carbyne with the PBE functional. For brevity, we will refer to the latter two basis sets as dcm[*C*_*diam*_]-TZVP, dcm[*C*_*graph*_]-TZVP, and dcm[*C*_*cby*_]-TZVP, respectively. Figure 3 shows a corresponding graphical representation of the radial component of some of the involved gaussians. The first striking effect observed is the overall contraction of exponents with respect to the molecular basis. This is not unexpected^15,19^ and is to be ascribed to the higher density of atoms in the solid-state phase.

**Table 1:** Uncontracted Gaussian Exponents for Different Carbon TZVP Basis Sets^b^

	def2^30^	pob^15^	pob-rev2^17^	dcm[*C*_*diam*_]^a^	dcm[*C*_*graph*_]^a^	dcm[*C*_*cby*_]^a^
s	0.5770	0.4941	0.4941	2.7288	1.0961	1.1383
	0.2297	0.1644	0.1644	0.7083	0.5911	0.6557
	0.0952			0.2754	0.2374	0.2323
p	0.2889	0.5662	0.5662	0.6187	0.3387	0.2857
	0.1006	0.2674	0.1973	0.2713	0.1594	0.0906
d	1.0970	0.8792	0.5792	2.0114	1.2502	1.3095
	0.3180			0.6265	0.7194	0.6132
f	0.7610			1.0624	0.7067	1.1330

a Present work.

b dcm[*C*_*diam*_]-TZVP, dcm[*C*_*graph*_]-TZVP, and dcm[*C*_*cby*_]-TZVP refer to our basis set optimized by BDIIS with the PBE functional in diamond, graphene, and carbyne, respectively.

The outermost *p*-type function shows probably the most significant difference between diamond and graphene. Such difference is due both to the different chemical bonding (*sp*-hybridization) and atomic density–graphene is a 2D system surrounded by vacuum in the third dimension. This vacuum offers more space for the Gaussian functions to expand and at the same time requires more extended functions to cover that empty space. Such interpretation is corroborated by the example of the 1D carbyne chain basis dcm[*C*_*cby*_]-TZVP, which features an even more diffuse *p*-shell. We take the opportunity here to remind that–conversely to plane-waves–in an atom-centered Gaussian-based approach the true 2D and 1D periodicity is possible, hence the vacuum in the nonperiodic directions is a *true* vacuum. The effect of the reduced dimensionality is, however, partly counterbalanced by a progressively shorter carbon–carbon distance that is 2.92 Å in diamond, 2.69 Å in graphene, and 2.39/2.46 Å in carbyne due to the different hybridization of the carbon atom in the three compounds. The more diffuse *p*-function is responsible for the failed convergence when using the graphene dcm[*C*_*graph*_]-TZVP basis set in diamond (Table 2). Also *d*- and *f*-type functions have a somewhat different spread in the two systems, showing that quadrupole and octupole interactions act differently in the two allotropes.

**Table 2:** Total Energies at the DFT/PBE Level for Diamond and Graphene as Computed with Different Triple-ζ Basis Sets^b^

*E*_*TOT*_^*PBE*^	pob^15^	pob-rev2^17^	dcm[*C*_*diam*_]^a^	dcm[*C*_*graph*_]^a^	dcm[*C*_*cby*_]^a^
diamond	–76.157894	–76.154752	**-76.161457**		
graphene	–76.155441	–76.158920	–76.158342	**-76.169383**	
carbyne	–76.072706	–76.086918	–76.073559	–76.096273	**-76.099140**

a This work.

b Energies in E_*h*_. Energies for the consistently optimized basis sets are reported in bold.

In Table 2 we report some total energies obtained at the DFT/PBE level: in addition to dcm-TZVP and pob-TZVP bases, the dcm[*C*_*diam*_]-TZVP basis was also tested in graphene and the dcm[*C*_*graph*_]-TZVP in diamond. From Table 2, we see that the energies relative to the proper dcm bases are lower by about 0.014 E_*h*_ than the pob- ones. On the other hand, swapping the two dcm-TZVP bases led to an energy similar to (though still lower than) that of pob-TZVP[G] while the more diffuse dcm[*C*_*graph*_]-TZVP turned out to be unusable in the more closely packed diamond lattice, leading to linear dependencies, numerical instabilities, and no possible SCF convergence in the end. The same happened when we tried to use the unmodified original molecular def2-TZVP basis sets, with the sole exception of carbyne thanks to its one-dimensional extension. The corresponding energy is −76.098306 E_*h*_, about 1 mE_*h*_ higher than our dcm[*C*_*cby*_]-TZVP result.

The optimization carried out with the BFGS algorithm leads to a very similar basis set for graphene, yielding essentially the same energy. For diamond, a significantly different result is obtained with respect to the BDIIS one reported in Table 1 with an energy that is 68 μE_*h*_ higher (exponents are 1.0163; 0.6367; 0.2392 for valence *s*-functions, 0.5646; 0.2728 for *p*-functions, 1.0242; 0.5957 for *d*-functions, and 0.8239 for the *f*-function).

#### 3.2.2 Crystalline NaCl, Na, and Cl_2_

Let us now compare the optimal basis set obtained with the PBE0 functional for three bulk structures with very different chemical bonding, namely: metallic Na, molecular Cl_2_, and ionic rocksalt NaCl, whose electronic charge densities are reported in Figure 1. As discussed in the Introduction, the significantly different features in the electronic structure expectedly require a different support and hence a specific basis set. The geometries adopted are fully reported in the Supporting Information and have been obtained from experimental references in the literature.^47−49^

In Table 3 we see that for the Cl_2_ molecular crystal, not unexpectedly, the original def2 basis set undergoes very little modifications when optimized in the solid. Actually, it performs much better than the pob-TZVP basis (see Table 5) with the total energy being 0.1 Ha lower. The removal of the outermost *p*-function in the pob basis sets leads to an overall decrease of the exponents of the remaining functions that partly compensates the contribution to the total energy of the missing function. If one includes the outermost *p*-function from the dcm basis set, a further energy lowering of 13 and 17 mE_*h*_ is observed for the pob and pob-rev2 basis set, respectively. However, this is not enough to reach the final energy of solid Cl_2_ as obtained with the optimized dcm basis set thus showing the crucial role of the outermost *p*-function.

**Table 3:** Gaussian Exponents for Different Cl TZVP Basis Sets^b^

	def2	pob^15^	pob-rev2^17^	dcm[Cl_2_]^a^	dcm[NaCl]^a^
s	0.5023	0.4499	0.4499	0.5075	0.5724
	0.1796	0.1364	0.1364	0.1831	0.2312
p	2.9433	2.8015	2.8015	2.8983	2.9386
	1.0405	0.7396	0.7896	1.1044	1.1903
	0.3846	0.2106	0.2106	0.4092	0.4697
	0.1307			0.1365	0.1747
d	0.3390	0.2373	0.2373	0.3326	0.2838
f	0.7060			0.5990	0.6898

a This work.

b The dcm-variants were optimized with the PBE0 functional.

The dcm[NaCl] basis for Cl, optimized in the rocksalt structure, features significantly more contracted exponents, as far as *s*- and *p*-functions are concerned, while the *d* exponent becomes more diffuse. As reported in Table 4, a stronger contraction is observed in exponents of the *s*-type orbitals in going from the molecular def2 to the bulk metal and then the ionic NaCl. In this case we had to remove the most diffuse *p*-function (0.03 au) in order to ensure convergence, but at difference with the pob-TZVP case, we were able to keep all the *d*-functions in.

**Table 4:** Gaussian Exponents for Different Na TZVP Basis Sets^b^

	def2	pob^15^	pob-rev2^17^	dcm[Na]^a^	dcm[NaCl]^a^
s	0.0500	0.6746	0.4246	0.2127	0.3436
	0.0193	0.1006	0.1205	0.0782	0.0828
p	0.4174	0.4009	0.4009	0.4034	0.4023
	0.0910	0.1007	0.1207	0.0851	0.0981
	0.0300				
d	2.6090	1.0463	0.3053	2.6086	2.6074
	0.4300			0.4337	0.4040
	0.1000			0.1115	0.0985

a This work.

b The dcm-variants were optimized with the PBE0 functional.

As shown in Table 5it can be seen that in all cases dcm- energies are significantly lower than pob- ones, and quite surprisingly the dcm[Cl_2_]-TZVP and dcm[Na]-TZVP basis sets seem to perform well also in the ionic case.

**Table 5:** Total Energies at the DFT/PBE0 Level for Na, Cl_2_, and NaCl as Computed with Different TZVP Basis Sets^b^

*E*_*TOT*_^*PBE*0^	pob^15^	pob-rev2^17^	dcm[Na]^a^	dcm[Cl_2_]^a^	dcm[NaCl]^a^
Na	–162.202291	–162.198836	**-162.210106**		–162.209707
Cl_2_	–1840.065404	–1840.052238		**-1840.186164**	–1840.175452
NaCl	–622.394522	–622.392474	–622.405254		**-622.405810**

a This work.

b Energies in E_*h*_. Energies for the consistently optimized basis sets are reported in bold.

Such basis sets effects are also reflected in geometry optimizations. In Table 6 we report the optimized lattice parameters obtained with the different basis sets. It is seen that the dcm-basis sets lead in all cases to an expanded volume with respect to the pob- ones and in the case of Na and NaCl also to a better agreement with experiment at the PBE0 level. In the molecular crystal Cl_2_, dispersion effects act as a key role, hence the plain PBE0 leads to an excessively expanded volume when the dcm[Cl_2_] basis is used, while the introduction of -D3 dispersion correction restores a more correct description. It is reasonable to assume that the volume expansion associated with the dcm[Cl_2_] basis is related to a mitigation of BSSE effects–which usually act as spurious dispersion.

**Table 6:** Experimental^47−49^ and Calculated Lattice Parameters for Solid Na, Cl_2_, and NaCl, as Obtained with Different Basis Sets^b^

PBE0		pob^15^	pob-rev2^17^	dcm[Na]^a^	dcm[Cl_2_]^a^	dcm[NaCl]^a^	exp
Na	a	4.041 (−0.184)	3.957 (−0.268)	4.258 (0.033)			4.225
	a	6.211 (0.066)			6.634 (0.489)		6.145
Cl_2_	b	4.387 (−0.008)			4.683 (0.287)		4.395
	c	8.126 (−0.028)			8.549 (0.395)		8.154
NaCl	a	5.602(−0.038)	5.609(−0.031)			5.644(0.004)	5.640

PBE0-D3		pob^15^	pob-rev2^17^	dcm[Na]^a^	dcm[Cl_2_]^a^	dcm[NaCl]^a^	exp
Cl_2_	a	6.011 (−0.134)	6.034 (−0.111)		6.175 (0.030)		6.145
	b	4.224 (−0.171)	4.255 (−0.140)		4.307 (−0.088)		4.395
	c	8.013 (−0.140)	8.021 (−0.133)		8.239 (0.085)		8.154

a This work.

b The difference with respect to the experimental reference is reported in round brackets.

### 3.3 Use of Large, Extended Basis Sets

**Figure 4:**
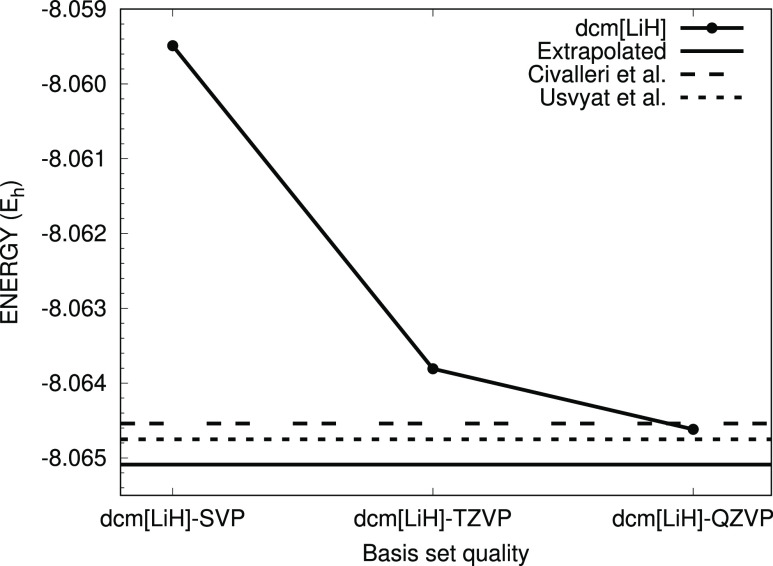
Energy of bulk LiH at the HF level: a comparison of our dcm-XVZP basis set results. Extrapolated CBS limit and literature data (Civalleri et al.^24^ and Usvyat et al.^26^).

Solid LiH is a rather standard benchmark for methods assessment in the solid state. Recently^24,26^ lithium hydride has been used as a benchmark for estimating the Hartree–Fock basis set limit compared with results from different approaches.^23,50,51^ The case of LiH, similarly as NaCl, poses certain difficulties since standard molecular basis sets are designed for neutral atoms, not ions, hence inapplicable to bulk ionic crystals without modification. We optimized the basis set series def2-SVP/def2-TZVP/def2-QZVP with our BDIIS algorithm, obtaining the corresponding dcm[LiH]-SVP/dcm[LiH]-TZVP/dcm[LiH]-QZVP. The corresponding Hartree–Fock total energy values are −8.059489, −8.063808, and −8.064618 E_*h*_, respectively. In Figure 4 we compare such energies with previous data from the literature also obtained with the CRYSTAL code. It is seen that with the quadruple basis we reach a value that is very close to that of ref (26) (i.e., −8.06475 E_*h*_), where a much larger basis set was used. This last result was already close to the CBS limit compared to methods employing different basis set types.^26^ If the dcm-TZVP and dcm-QZVP total energies are used to estimate the HF complete basis set (CBS) limit by using a two-point extrapolation scheme based on an exponential formula, a value of −8.065089 is attained. Notably, this energy limit is even lower than the one reached by Usvyat and co-workers^26^ by 0.3 mE_*h*_. When using the CBS energy for the atoms,^52^ the cohesive energy is then −3.60 eV in very good agreement with results from different theoretical approaches.^23,50,51^

Similarly, we have optimized a quadruple-ζ basis for diamond and graphene. The original def2-QZVP basis does not allow convergence in either case, while with the basis sets as reported in Table 7 the energies of −76.165178 and −76.174386 E_*h*_ are obtained for the two systems at the PBE level. The latter value we believe to be close to basis set completeness. Extrapolation to the CBS limit leads to a value of −76.167396 and −76.177298 E_*h*_, respectively.

**Table 7:** Exponents Comparison of Gaussian Basis Sets^b^

	def2-QZVP	dcm[*C*_*diam*_]-QZVP^a^	dcm[*C*_*graph*_]-QZVP^a^
s	5.2404	6.2060	5.2404
	2.2905	3.3250	2.3278
	0.6967	1.0952	1.0461
	0.2760	0.6304	0.5218
	0.1074	0.3436	0.2004
p	0.4605	0.8740	0.4500
	0.1894	0.5426	0.3287
	0.0760	0.1832	0.1249
d	1.8480	1.9639	1.9130
	0.6490	0.9684	0.8630
	0.2280	0.5478	0.4526
f	1.4190	1.5109	1.4143
	0.4850	0.7423	0.5982
g	1.0110	1.1825	0.9931

a This work.

b Diamond and graphene cases for quadruple-ζ basis sets. Optimization carried out at the PBE level.

In Table 7 we report the reoptimized exponents with respect to def2-QZVP basis sets–all other functions are the same as in the molecular basis set. It is worth noting that *g*-type functions were also included in the basis set as they were recently made available in the development version of the Crystal code.^53^

BSSE effects are reduced much more considerably by the increasing of the basis set quality, rather than by the optimization of the exponents, so that BSSE is quite similar for pob- or dcm- basis sets.

For diamond, we have also calculated the Hartree–Fock CBS limit by using the dcm-TZVP and dcm-QZVP basis sets. Computed total energies are −75.772638 and −75.774752 E_*h*_, respectively. The adopted extrapolation scheme leads to a HF CBS limit of −75.775983 E_*h*_ that corresponds to a cohesive energy for diamond^52^ of −10.58 eV, very close to a previously reported HF value of −10.56 eV.^54^ Interestingly, results show that the basis sets optimized with the PBE functional can also be used for HF even if a tighter setting of the computational parameters is required (see the Supporting Information).

**Figure 5:**
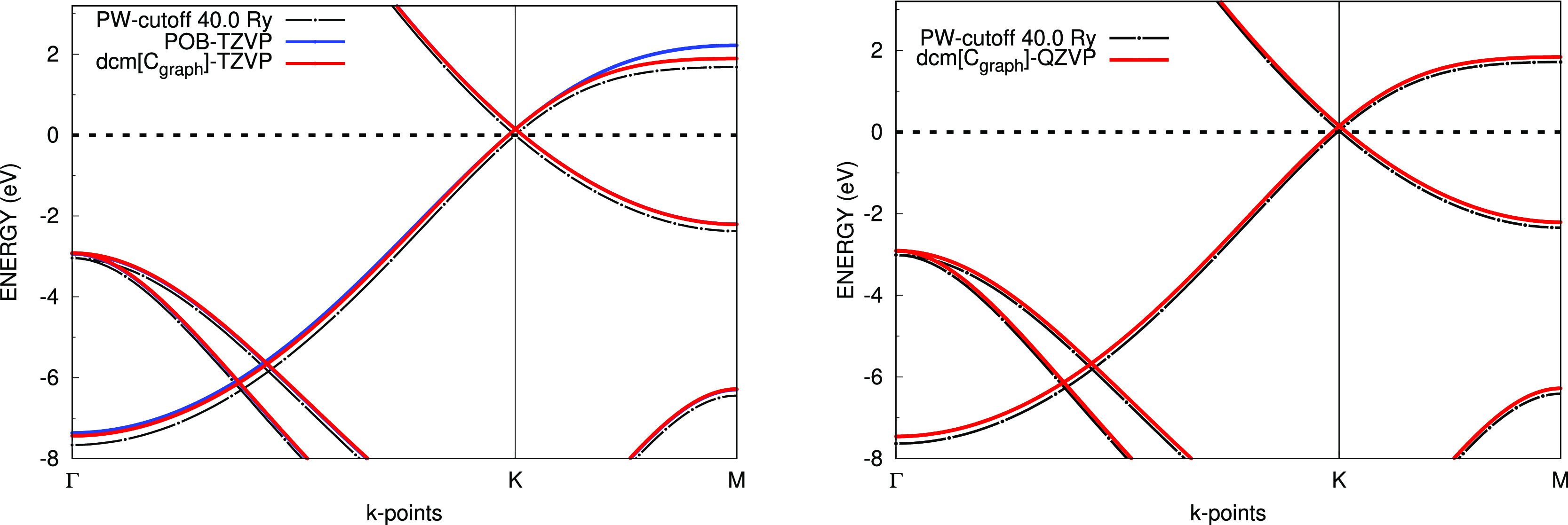
Band structure comparison between plane waves (Quantum Espresso) and CRYSTAL in the graphene case with dcm[*C*_*graph*_] and POB basis sets with PBE as the DFT functional.

In Figure 5 we compare, for graphene, the electronic band structure computed with Gaussian basis sets with the bands for the same systems as obtained from a plane wave code^55^ using a considerably high cutoff. It is evident that the bands at the triple-ζ level are different from the reference ones, specially in the Γ and *M* points of the Brillouin zone. Nevertheless, the dcm[*C*_*graph*_]-TZVP performs better than the pob-TZVP. A considerably better agreement is attained by using the dcm[*C*_*graph*_]-QZVP basis (right panel of Figure 5). We believe this is strong evidence of the possibility of reaching converged results with Gaussian basis sets and the effectiveness of a system-specific optimization scheme.

## 4 Conclusions

In the present work, we have developed a basis set optimizer based on the DIIS algorithm that minimizes the total energy of the system constrained to keep the condition number of the overlap matrix as small as possible in a similar approach as proposed by VandeVondele et al.^40^ The latter constraint acts as a pivot in the optimization of the basis set and prevents the lowest exponents of the basis set to decrease too much thus reducing the risk of linear dependency and numerical instability. This is particularly important in solid-state calculations where the use of atom-centered diffuse functions is more delicate and sometime useless.

We have then shown that the proposed method is quite effective for solid-state calculations and allows for an easy optimization of basis sets not only of triple-ζ quality but even of quadruple-ζ size. Furthermore, we have demonstrated that the BDIIS method can be used to obtain basis sets for solids of consistent quality as molecules without pruning the original basis sets. Results for simple solids as diamond and graphene for which the definition of an appropriate and system-consistent basis set is uglily difficult are very promising. Also, the possibility of employing basis sets specifically calibrated on a given system allowed us to easily reach the HF complete basis set limit for LiH which has been a long debated issue and for diamond.

While reasonable questions can be raised about the transferability of such optimized basis sets from one method to another, we have seen that a basis set optimized, say, with PBE is very close to convergence when inserted in HF or PBE0. For our diamond test case the energy with such basis was only a few μE_*h*_ away from the minimum when transferred from one method to another.

The evidence of the excellent performance of the BDIIS method paves the way for a careful definition of system-specific basis sets, as a viable alternative to all-purpose basis sets. Nevetheless, it could be employed for a more extensive work that would permit the creation of all-purpose basis set families for a larger set of atomic species. Furthermore, the algorithm here described could be very useful to optimize basis sets for post-HF correlation methods^20,56^ as well as for response properties.^57−59^

## Supporting Information Available

Crystal17 inputs used to obtain reported results (PDF)
