# Supplementary material for: Gaussian Basis Sets for Crystalline Solids: All-Purpose Basis Set Libraries vs System-Specific Optimizations
Source: J Chem Theory Comput. 2020 Mar 26;16(4):2192–201. doi: 10.1021/acs.jctc.9b01004 (PMC7997400; doi:10.1021/acs.jctc.9b01004)
Supplement: Supplementary file 1 — ct9b01004_si_001.pdf [file ct9b01004_si_001.pdf]

Supplementary Material for: [Gaussian Basis sets for Crystalline Solids: All-Purpose Basis Set Libraries Vs System–Specific Optimizations]

Loredana Edith Daga, Bartolomeo Civalleri, and Lorenzo Maschio*

Dipartimento di Chimica, Università di Torino and NIS (Nanostructured Interfaces and Surfaces) Centre, Via P. Giuria 5, 10125 Torino, Italy

E-mail: lorenzo.maschio@unito.it

This document explicitly reports most of the CRYSTAL17 inputs used to obtain the results reported in the paper. The basis set in the inputs are the dcm-XZVP obtained by using the BDIIS optimizer. The shells labelled by an asterisk are those that have been optimized.

1 Diamond - triple ζ

```
DIAMOND-TZ-PBE
CRYSTAL
0 0 1
227
3.56679
1
6   0. 0. 0.
END
  6 11
0 0  6  2. 1.
   1.35753496820E+04   2.22458143520E-04
   2.03523336800E+03   1.72327382520E-03
   4.63225623590E+02   8.92557153140E-03
   1.31200195980E+02   3.57279845020E-02
   4.28530158910E+01   1.10762599310E-01
   1.55841857660E+01   2.42956276260E-01
0 0  2  2. 1.
   6.20671385080E+00   4.14402634480E-01
   2.57648965270E+00   2.37449686550E-01
0 0  1  0. 1. *
   2.72877227664E+00   1.00000000000E+00
0 0  1  0. 1. *
   7.08254466129E-01   1.00000000000E+00
0 0  1  0. 1. *
   2.75435053993E-01   1.00000000000E+00
```

```
0 2  4  2. 1.
   3.46972322440E+01   5.33336578050E-03
   7.95826228260E+00   3.58641090920E-02
   2.37808268830E+00   1.42158733290E-01
   8.14332081830E-01   3.42704718450E-01
0 2  1  0. 1. *
   6.18739070583E-01   1.00000000000E+00
0 2  1  0. 1. *
   2.71282775658E-01   1.00000000000E+00
0 3  1  0. 1. *
   2.01143808439E+00   1.00000000000E+00
0 3  1  0. 1. *
   6.26539832528E-01   1.00000000000E+00
0 4  1  0. 1. *
   1.06242225484E+00   1.00000000000E+00
99 0
END
DFT
PBE
END
MAXCYCLE
100
TOLINTEG
8 8 8 12 24
TOLDEE
8
SHRINK
8 8
END
```

2 Diamond - quadruple ζ

```
DIAMOND-QZ-PBE
CRYSTAL
0 0 1
227
3.56679
1
6   0. 0. 0.
END
  6 17
0 0  8  2. 1.
   6.70250710290E+04   3.87363085010E-05
   1.00399865380E+04   3.01079175750E-04
   2.28493169110E+03   1.57879180950E-03
   6.47141221300E+02   6.60870871950E-03
   2.11094723350E+02   2.33671232500E-02
   7.61776438620E+01   7.04207168980E-02
   2.96338391630E+01   1.73603449530E-01
   1.21877850810E+01   3.22923056480E-01
0 0  2  2. 1.
   5.30260062990E+01   7.48974044920E-02
   1.52585027760E+01   7.61362209830E-01
0 0  1  0. 1. *
   6.20602927512E+00   1.00000000000E+00
0 0  1  0. 1. *
   3.32497590179E+00   1.00000000000E+00
0 0  1  0. 1. *
   1.09521378286E+00   1.00000000000E+00
0 0  1  0. 1. *
   6.30358128105E-01   1.00000000000E+00
0 0  1  0. 1. *
   3.43646141101E-01   1.00000000000E+00
```

```
0 2  5  2. 1.
   1.05125550820E+02   8.46475538440E-04
   2.48844610660E+01   6.62740385340E-03
   7.86372308260E+00   3.01203904190E-02
   2.84070018350E+00   9.99514354760E-02
   1.12271373350E+00   2.38262992820E-01
0 2  1  0. 1. *
   8.74041963051E-01   1.00000000000E+00
0 2  1  0. 1. *
   5.42561494412E-01   1.00000000000E+00
0 2  1  0. 1. *
   1.83247854025E-01   1.00000000000E+00
0 3  1  0. 1. *
   1.96394180988E+00   1.00000000000E+00
0 3  1  0. 1. *
   9.68369087225E-01   1.00000000000E+00
0 3  1  0. 1. *
   5.47772457307E-01   1.00000000000E+00
0 4  1  0. 1. *
   1.51085897443E+00   1.00000000000E+00
0 4  1  0. 1. *
   7.42254560983E-01   1.00000000000E+00
0 5  1  0. 1. *
   1.18250314805E+00   1.00000000000E+00
99 0
END
DFT
PBE
END
MAXCYCLE
100
TOLINTEG
8 8 8 12 24
```

```
TOLDEE
8
SHRINK
8 8
NOBIPOLA
END
```

3 Graphene - triple ζ

```
GRAPHENE-TZ-PBE
SLAB
77
2.47
1
6  -0.33333333333 0.33333333333 0.
END
  6 11
0 0  6  2. 1.
   1.35753496820E+04   2.22458143520E-04
   2.03523336800E+03   1.72327382520E-03
   4.63225623590E+02   8.92557153140E-03
   1.31200195980E+02   3.57279845020E-02
   4.28530158910E+01   1.10762599310E-01
   1.55841857660E+01   2.42956276260E-01
0 0  2  2. 1.
   6.20671385080E+00   4.14402634480E-01
   2.57648965270E+00   2.37449686550E-01
0 0  1  0. 1. *
   1.09605489209E+00   1.00000000000E+00
0 0  1  0. 1. *
   5.91106934956E-01   1.00000000000E+00
```

```
0 0  1  0. 1. *
   2.37383807053E-01   1.00000000000E+00
0 2  4  2. 1.
   3.46972322440E+01   5.33336578050E-03
   7.95826228260E+00   3.58641090920E-02
   2.37808268830E+00   1.42158733290E-01
   8.14332081830E-01   3.42704718450E-01
0 2  1  0. 1. *
   3.38693223652E-01   1.00000000000E+00
0 2  1  0. 1. *
   1.59434147140E-01   1.00000000000E+00
0 3  1  0. 1. *
   1.25021363036E+00   1.00000000000E+00
0 3  1  0. 1. *
   7.19402508165E-01   1.00000000000E+00
0 4  1  0. 1. *
   7.06699996366E-01   1.00000000000E+00
99 0
END
DFT
PBE
XLGRID
END
SCFDIR
FMIXING
30
LDREMO
1
MAXCYCLES
100
TOLINTEG
8 8 8 8 16
TOLDEE
```

```
8
SHRINK
64 64
END
```

4 Graphene - quadruple ζ

```
GRAPHENE-QZ-PBE
SLAB
77
2.47
1
6 -0.33333333333 0.33333333333 0.
END
6 17
0 0  8  2. 1.
   6.70250710290E+04   3.87363085010E-05
   1.00399865380E+04   3.01079175750E-04
   2.28493169110E+03   1.57879180950E-03
   6.47141221300E+02   6.60870871950E-03
   2.11094723350E+02   2.33671232500E-02
   7.61776438620E+01   7.04207168980E-02
   2.96338391630E+01   1.73603449530E-01
   1.21877850810E+01   3.22923056480E-01
0 0  2  2. 1.
   5.30260062990E+01   7.48974044920E-02
   1.52585027760E+01   7.61362209830E-01
0 0  1  0. 1. *
   5.24039574640E+00   1.00000000000E+00
0 0  1  0. 1. *
   2.32777405666E+00   1.00000000000E+00
0 0  1  0. 1. *
```

```
   1.04613233770E+00   1.00000000000E+00
0 0  1  0. 1. *
   5.21832251512E-01   1.00000000000E+00
0 0  1  0. 1. *
   2.00431897163E-01   1.00000000000E+00
0 2  5  2. 1.
   1.05125550820E+02   8.46475538440E-04
   2.48844610660E+01   6.62740385340E-03
   7.86372308260E+00   3.01203904190E-02
   2.84070018350E+00   9.99514354760E-02
   1.12271373350E+00   2.38262992820E-01
0 2  1  0. 1. *
   4.50031980659E-01   1.00000000000E+00
0 2  1  0. 1. *
   3.28748485841E-01   1.00000000000E+00
0 2  1  0. 1. *
   1.24896018560E-01   1.00000000000E+00
0 3  1  0. 1. *
   1.91295701725E+00   1.00000000000E+00
0 3  1  0. 1. *
   8.63019243591E-01   1.00000000000E+00
0 3  1  0. 1. *
   4.52624895593E-01   1.00000000000E+00
0 4  1  0. 1. *
   1.41426143665E+00   1.00000000000E+00
0 4  1  0. 1. *
   5.98189433168E-01   1.00000000000E+00
0 5  1  0. 1. *
   9.93072428023E-01   1.00000000000E+00
99 0
END
DFT
PBE
```

```
XLGRID
END
SCFDIR
FMIXING
30
LDREMO
1
MAXCYCLES
100
TOLINTEG
8 8 8 8 16
TOLDEE
8
SHRINK
64 64
END
```

5 Carbyne - triple ζ

```
C_Chain
POLYMER
1
2.56620186
2
6   9.624791798260E-02 -1.258372661974E-18 -4.072637834921E-09
6  -3.962479179826E-01 -2.785734209432E-16  4.072638114753E-09
END
6 11
0 0 6 2. 1.
1.35753496820E+04 2.22458143520E-04
2.03523336800E+03 1.72327382520E-03
4.63225623590E+02 8.92557153140E-03
```

```
1.31200195980E+02 3.57279845020E-02
4.28530158910E+01 1.10762599310E-01
1.55841857660E+01 2.42956276260E-01
0 0 2 2. 1.
6.20671385080E+00 4.14402634480E-01
2.57648965270E+00 2.37449686550E-01
0 0 1 0. 1. *
 1.13833219587E+00   1.00000000000E+00
0 0 1 0. 1. *
 6.55714337901E-01   1.00000000000E+00
0 0 1 0. 1. *
 2.32302377804E-01   1.00000000000E+00
0 2 4 2. 1.
3.46972322440E+01 5.33336578050E-03
7.95826228260E+00 3.58641090920E-02
2.37808268830E+00 1.42158733290E-01
8.14332081830E-01 3.42704718450E-01
0 2 1 0. 1. *
 2.85725969158E-01   1.00000000000E+00
0 2 1 0. 1. *
 9.05806201973E-02   1.00000000000E+00
0 3 1 0. 1. *
 1.30952322712E+00   1.00000000000E+00
0 3 1 0. 1. *
 6.13241389114E-01   1.00000000000E+00
0 4 1 0. 1. *
 1.13300431880E+00   1.00000000000E+00
99 0
END
DFT
PBE
XLGRID
END
```

```
LDREMO
1
SCFDIR
LEVSHIFT
10 1
FMIXING
30
MAXCYCLES
100
TOLINTEG
8 8 8 8 16
TOLDEE
8
SHRINK
64 64
END
```

6 NaCl - triple ζ

```
NaCl_crystal-TZ-PBE0
CRYSTAL
0 0 0
225
5.6402
2
17 0.0 0.0 0.0
11 0.5 0.5 0.5
END
 11 11
0 0  7  2. 1.
   2.60411099270E+04   6.18063428110E-04
   3.90612685480E+03   4.77486044140E-03
```

```
   8.88974549930E+02   2.44716848290E-02
   2.51454979610E+02   9.47553949770E-02
   8.16501435120E+01   2.68674969200E-01
   2.89041584010E+01   4.79254754400E-01
   1.06257829320E+01   3.32485914690E-01
0 0  3  2. 1.
   5.37694101790E+01   1.95277318720E-02
   1.63082430250E+01   9.26480107940E-02
   2.37303841250E+00  -3.99386701720E-01
0 0  2  1. 1.
   9.57307726030E-01   1.64285953910E+00
   4.08064609590E-01   5.56925969660E-01
0 0  1  0. 1. *
   3.43569978393E-01   9.99992062637E-01
0 0  1  0. 1. *
   8.28104267544E-02   9.99700925116E-01
0 2  5  6. 1.
   1.38079799890E+02   5.79518919290E-03
   3.22327003930E+01   4.16208462510E-02
   9.98160753600E+00   1.62819168850E-01
   3.48220339280E+00   3.60117846470E-01
   1.22991346200E+00   4.48589798890E-01
0 2  1  0. 1. *
   4.02257637589E-01   1.00001437874E+00
0 2  1  0. 1. *
   9.81092534535E-02   1.00010780928E+00
0 3  1  0. 1. *
   2.60738659094E+00   1.00000000000E+00
0 3  1  0. 1. *
   4.03951011422E-01   1.00000000000E+00
0 3  1  0. 1. *
   9.84682729829E-02   1.00000000000E+00
 17 13
```

```
0 0  7  2. 1.
   6.95079909450E+04   5.43148974970E-04
   1.04261568800E+04   4.19904639610E-03
   2.37323340610E+03   2.15921416790E-02
   6.71564200710E+02   8.45988500940E-02
   2.18419997900E+02   2.47572497240E-01
   7.75722497140E+01   4.70169302280E-01
   2.88888152770E+01   3.74363707160E-01
0 0  3  2. 1.
   1.27105271850E+02   2.51821666030E-02
   3.93395829610E+01   1.07861124560E-01
   7.67406799890E+00  -2.74088215740E-01
0 0  2  2. 1.
   3.87456276300E+00   1.32138750140E+00
   1.83858325730E+00   6.86369553680E-01
0 0  1  0. 1. *
   5.72362924707E-01   1.00000000308E+00
0 0  1  0. 1. *
   2.31162823985E-01   9.99999997835E-01
0 2  5  6. 1.
   6.66504232840E+02   2.36326638360E-03
   1.57642416900E+02   1.88793003740E-02
   5.02625209780E+01   8.72063412730E-02
   1.85360781050E+01   2.52856129700E-01
   7.29405327770E+00   4.35071548200E-01
0 2  1  5. 1. *
   2.93863280310E+00   1.00000000143E+00
0 2  1  0. 1. *
   1.19026410565E+00   1.00000000000E+00
0 2  1  0. 1. *
   4.69704038156E-01   1.00000000000E+00
0 2  1  0. 1. *
   1.74720163208E-01   1.00000000000E+00
```

```
0 3  2  0. 1.
   4.61000000000E+00   2.00000000000E-01
   1.01100000000E+00   1.00000000000E+00
0 3  1  0. 1. *
   2.83798017576E-01   1.00000000000E+00
0 4  1  0. 1. *
   6.89848600893E-01   1.00000000000E+00
99 0
END
DFT
PBE0
END
TOLINTEG
8 8 8 8 16
TOLDEE
8
SHRINK
8 8
END
```

7 Na - triple ζ

```
Na_crystal-TZ-PBE0
CRYSTAL
0 0 0
229
4.2906
1
11 0. 0. 0.
END
 11 11
0 0  7  2. 1.
```

```
   2.60411099270E+04   6.18063428110E-04
   3.90612685480E+03   4.77486044140E-03
   8.88974549930E+02   2.44716848290E-02
   2.51454979610E+02   9.47553949770E-02
   8.16501435120E+01   2.68674969200E-01
   2.89041584010E+01   4.79254754400E-01
   1.06257829320E+01   3.32485914690E-01
0 0  3  2. 1.
   5.37694101790E+01   1.95277318720E-02
   1.63082430250E+01   9.26480107940E-02
   2.37303841250E+00  -3.99386701720E-01
0 0  2  1. 1.
   9.57307726030E-01   1.64285953910E+00
   4.08064609590E-01   5.56925969660E-01
0 0  1  0. 1. *
   2.12721167582E-01   9.99992062637E-01
0 0  1  0. 1. *
   7.82096876345E-02   9.99700925116E-01
0 2  5  6. 1.
   1.38079799890E+02   5.79518919290E-03
   3.22327003930E+01   4.16208462510E-02
   9.98160753600E+00   1.62819168850E-01
   3.48220339280E+00   3.60117846470E-01
   1.22991346200E+00   4.48589798890E-01
0 2  1  0. 1. *
   4.03415531731E-01   1.00001437874E+00
0 2  1  0. 1. *
   8.50539333916E-02   1.00010780928E+00
0 3  1  0. 1. *
   2.60862497919E+00   1.00000000000E+00
0 3  1  0. 1. *
   4.33739110192E-01   1.00000000000E+00
0 3  1  0. 1. *
```

```
   1.11542851169E-01   1.00000000000E+00
99 0
END
DFT
PBE0
END
TOLINTEG
8 8 8 8 16
TOLDEE
8
SHRINK
8 8
END
```

8 Cl_2 - triple ζ

```
Cl_crystal-TZ-PBE0
CRYSTAL
0 0 0
64
7.77875 4.34941 9.02754
8
17 0            0.40882      0.89848
17 0            0.09118      0.39848
17 0            0.90882      0.60152
17 0            0.59118      0.10152
17 0.50000      0.90882      0.89848
17 0.50000      0.59118      0.39848
17 0.50000      0.40882      0.60152
17 0.50000      0.09118      0.10152
END
 17 13
```

```
0 0  7  2. 1.
   6.95079909450E+04   5.43148974970E-04
   1.04261568800E+04   4.19904639610E-03
   2.37323340610E+03   2.15921416790E-02
   6.71564200710E+02   8.45988500940E-02
   2.18419997900E+02   2.47572497240E-01
   7.75722497140E+01   4.70169302280E-01
   2.88888152770E+01   3.74363707160E-01
0 0  3  2. 1.
   1.27105271850E+02   2.51821666030E-02
   3.93395829610E+01   1.07861124560E-01
   7.67406799890E+00  -2.74088215740E-01
0 0  2  2. 1.
   3.87456276300E+00   1.32138750140E+00
   1.83858325730E+00   6.86369553680E-01
0 0  1  0. 1.
   5.07498154767E-01   1.00000000308E+00
0 0  1  0. 1.
   1.83127456586E-01   9.99999997835E-01
0 2  5  6. 1.
   6.66504232840E+02   2.36326638360E-03
   1.57642416900E+02   1.88793003740E-02
   5.02625209780E+01   8.72063412730E-02
   1.85360781050E+01   2.52856129700E-01
   7.29405327770E+00   4.35071548200E-01
0 2  1  5. 1.
   2.89826632488E+00   1.00000000143E+00
0 2  1  0. 1.
   1.10442102086E+00   1.00000000000E+00
0 2  1  0. 1.
   4.09232170559E-01   1.00000000000E+00
0 2  1  0. 1.
   1.36452167048E-01   1.00000000000E+00
```

```
0 3  2  0. 1.
   4.61000000000E+00   2.00000000000E-01
   1.01100000000E+00   1.00000000000E+00
0 3  1  0. 1.
   3.32581229403E-01   1.00000000000E+00
0 4  1  0. 1.
   5.98970631104E-01   1.00000000000E+00
99 0
END
DFT
PBE0
END
TOLDEE
8
TOLINTEG
8 8 8 8 16
SHRINK
8 8
END
```

9 LiH - SVP

```
LiH-SVP-HF
CRYSTAL
0 0 0
225
4.084
2
3  0.0  0.0  0.0
1  0.5  0.5  0.5
END
  3  5
```

```
0 0  5  2. 1.
   2.66277855160E+02   6.49201503250E-03
   4.00697834470E+01   4.77478632150E-02
   9.05599443890E+00   2.02687961110E-01
   2.45030090510E+00   4.86065748170E-01
   7.22095718550E-01   4.36269779550E-01
0 0  1  1. 1. *
   9.22176144583E-01   1.00000000000E+00
0 0  1  0. 1. *
   3.26710606911E-01   1.00000000000E+00
0 2  2  0. 1.
   1.45000000000E+00   2.58600000000E-01
   3.00000000000E-01   1.00000000000E+00
0 2  1  0. 1. *
   2.44108651001E-01   1.00000000000E+00
  1  3
0 0  3  1. 1.
   1.30107010000E+01   1.96821580000E-02
   1.96225720000E+00   1.37965240000E-01
   4.44537960000E-01   4.78319350000E-01
0 0  1  0. 1. *
   1.12026126213E-01   1.00000000000E+00
0 2  1  0. 1. *
   7.04259338539E-01   1.00000000000E+00
99 0
END
TOLINTEG
8 8 8 15 30
SHRINK
8 8
FMIXING
50
TOLDEE
```

```
10
END
```

10 LiH - triple ζ

```
LiH-TZ-HF
CRYSTAL
0 0 0
225
4.084
2
3  0.0  0.0  0.0
1  0.5  0.5  0.5
END
  3  8
0 0  6  2. 1.
   6.26926280100E+03   2.05409688260E-04
   9.40316124310E+02   1.59165540890E-03
   2.14221075280E+02   8.28698297070E-03
   6.07598401840E+01   3.38563742490E-02
   1.99151520320E+01   1.11032258760E-01
   7.31715097970E+00   2.74493833290E-01
0 0  2  1. 1.
   2.97246742160E+00   2.37924564110E-01
   1.26398523140E+00   3.07654119240E-01
0 0  1  0. 1. *
   7.80051836987E-01   1.00000000000E+00
0 0  1  0. 1. *
   3.56751423380E-01   1.00000000000E+00
0 0  1  0. 1. *
   1.32441081592E-01   1.00000000000E+00
0 2  1  0. 1. *
```

```
   5.32524694781E-01   1.00000000000E+00
0 2  1  0. 1. *
   1.24297352468E-01   1.00000000000E+00
0 2  1  0. 1. *
   3.32168908872E+00   1.00000000000E+00
  1  4
0 0  3  1. 1.
   3.40613410000E+01   6.02519780000E-03
   5.12357460000E+00   4.50210940000E-02
   1.16466260000E+00   2.01897260000E-01
0 0  1  0. 1. *
   7.93610197287E-01   1.00000000000E+00
0 0  1  0. 1. *
   2.38040797833E-01   1.00000000000E+00
0 2  1  0. 1. *
   7.51152930456E-01   1.00000000000E+00
99 0
END
TOLINTEG
10 10 10 15 30
SHRINK
8 8
FMIXING
50
TOLDEE
10
NOBIPOLA
END
```

11 LiH - quadruple ζ

```
LiH-QZ-HF
```

```
CRYSTAL
0 0 0
225
4.084
2
3  0.0  0.0  0.0
1  0.5  0.5  0.5
LATVEC
100000
END
  1 10
0 0  4  1. 1.
   1.90691690000E+02   7.08151670000E-04
   2.86055320000E+01   5.46788270000E-03
   6.50959430000E+00   2.79666050000E-02
   1.84124550000E+00   1.07645380000E-01
0 0  1  0. 1. *
   6.39685855118E-01   1.00000000000E+00
0 0  1  0. 1. *
   2.72584652976E-01   1.00000000000E+00
0 0  1  0. 1. *
   1.44295727123E-01   1.00000000000E+00
0 2  1  0. 1. *
   2.29203807546E+00   1.00000000000E+00
0 2  1  0. 1. *
   8.37965510305E-01   1.00000000000E+00
0 2  1  0. 1. *
   2.95979305342E-01   1.00000000000E+00
0 3  1  0. 1. *
   2.06195543104E+00   1.00000000000E+00
0 3  1  0. 1. *
   6.61486854173E-01   1.00000000000E+00
0 4  1  0. 1. *
```

```
   1.39686108048E+00   1.00000000000E+00
  3 13
0 0  9  2. 1.
   1.48539770850E+04   4.27113888150E-05
   2.22522364770E+03   3.32353108000E-04
   5.04887390080E+02   1.75184366490E-03
   1.42458475480E+02   7.34779958500E-03
   4.63155995800E+01   2.58998376830E-02
   1.66553354740E+01   7.66706824310E-02
   6.43311861990E+00   1.82760757650E-01
   2.60270438580E+00   3.26554340380E-01
   1.08972454050E+00   3.70004298280E-01
0 0  2  1. 1.
   4.42365959710E+00   1.11209879210E-01
   1.23563949900E+00   7.99873358620E-01
0 0  1  0. 1. *
   1.47258539716E+00   1.00000000000E+00
0 0  1  0. 1. *
   9.61687138188E-01   1.00000000000E+00
0 0  1  0. 1. *
   5.44705804681E-01   1.00000000000E+00
0 0  1  0. 1. *
   2.59775255362E-01   1.00000000000E+00
0 2  3  0. 1.
   3.26051092060E+00   8.65047490230E-03
   6.50030431150E-01   4.76141237360E-02
   1.69416710730E-01   2.10011380000E-01
0 2  1  0. 1. *
   3.83906409071E-01   1.00000000000E+00
0 2  1  0. 1. *
   1.98706020944E-01   1.00000000000E+00
0 2  1  0. 1. *
   3.72683460316E+00   1.00000000000E+00
```

```
0 3  1  0. 1. *
   3.33113752884E-01   1.00000000000E+00
0 3  1  0. 1. *
   3.68482801264E-01   1.00000000000E+00
0 4  1  0. 1. *
   1.75294244744E-01   1.00000000000E+00
99 0
END
TOLINTEG
10 10 10 40 160
SHRINK
12 12
NOBIPOLA
LDREMO
1
FMIXING
96
THREDIIS
2
TOLDEE
10
ILASIZE
20000
END
```
